# Losartan API and Nitrosamines Impurities—Stability Profile Investigation Using HPLC, LC‐MS, and In Silico Assessment

**DOI:** 10.1002/jssc.70438

**Published:** 2026-05-08

**Authors:** Paulo Roberto Rodrigues Martini, Lilian Fanfa Machioli, Bruno Pereira dos Santos, Tiago Franco de Oliveira, Fávero Reisdorfer Paula, Clésio Soldateli Paim, Andreas Sebastian Loureiro Mendez

**Affiliations:** ^1^ Postgraduation Program in Pharmaceutical Sciences Federal University of Rio Grande Do Sul (UFRGS) Porto Alegre Rio Grande do Sul Brazil; ^2^ Postgraduation Program in Health Sciences Federal University of Health Sciences of Porto Alegre (UFCSPA) Porto Alegre Rio Grande do Sul Brazil; ^3^ Postgraduation Program in Pharmaceutical Sciences Federal University of Pampa (UNIPAMPA) Uruguaiana Rio Grande do Sul Brazil

**Keywords:** degradation products, HPLC, in silico modeling, LC‐MS, losartan, nitrosamines, stability

## Abstract

Nitrosamines are potent genotoxic impurities with well reported mutagenic and carcinogenic effects. Their presence has been detected in several pharmaceutical products among them losartan, an angiotensin II receptor blocker. Considering the risks already reported for these impurities, the present study aimed to investigate the influence of four common nitrosamines—N‐nitrosodimethylamine (NDMA), N‐nitrosodiethylamine (NDEA), N‐nitrosodiisopropylamine (NDIPA), and N‐nitrosodibutylamine (NDBA)—in the stability profile of losartan API. Quantitative assay was conducted through HPLC and LC‐MS, with focus on monitoring degradation rate and degradation products under influence of nitrosamines. Predictive data by in silico investigation employing Zeneth Nexus and Spartan were simultaneously studied, considering degradation products, fragmentation pathway and nitrosamines reactivity. The drug residual content showed variability depending on the nitrosamine evaluated and the stressing condition applied. From photolysis, for example the residual content ranged 80%–90%, with a greater decomposition in samples containing nitrosamines. A greater decomposition was also observed in oxidative degradation, with exception for samples containing NDMA. Acid and basic media caused a significant decomposition, with the residual losartan content in a range of 41%–52% and 28%–51%, respectively. From LC‐MS analyzes, these impurities were mostly not detected in the degraded samples, suggesting their consumption during the reaction due to their reactivity. A protective effect from nitrosamines can be also reported possibly due to their reactivity against the stressing factor. Seven degradation products were structurally proposed by LC‐MS at *m*/*z* 449, *m*/*z* 447, *m*/*z* 366, *m*/*z* 338, *m*/*z* 274, *m*/*z* 391, and *m*/*z* 341, some of them predicted computationally by Zeneth.

## 1 Introduction

**FIGURE 1:**
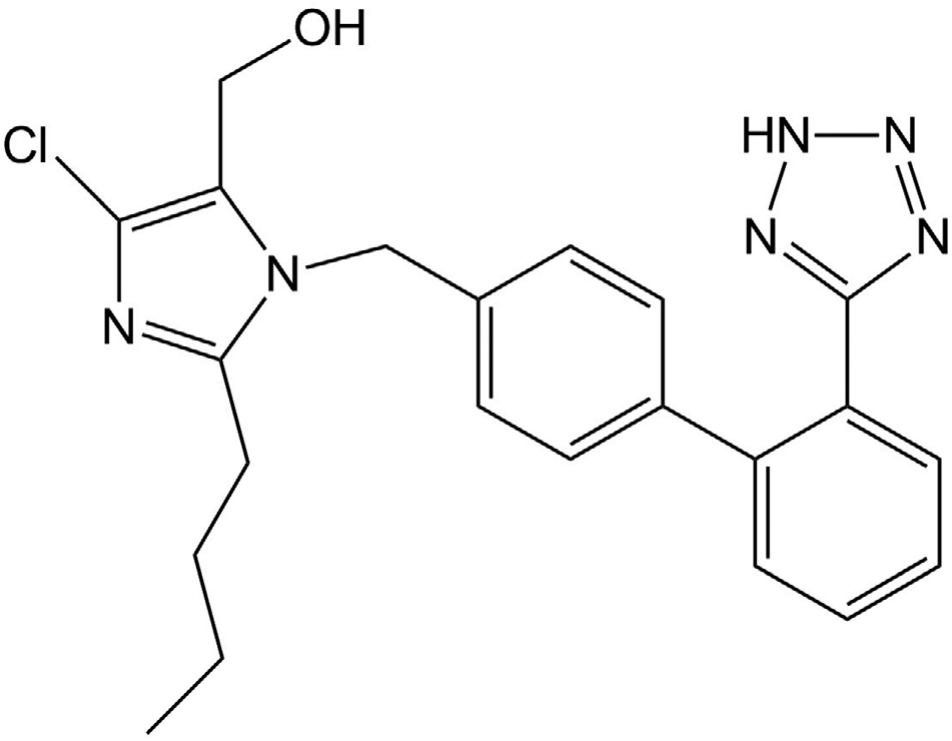
Chemical structure of losartan.

Losartan potassium is a salt of 2‐*n*‐butyl‐4‐chloro‐1‐[[2′‐(1H‐tetrazol‐5‐yl)biphenyl‐4‐yl]methyl] (Figure 1). It belongs to a class of medicines known as angiotensin II receptor blockers (ARBs) also described as selective antagonists of angiotensin II receptor type 1 AT_1_ [1], and referred to as “sartans” [2]. This drug is commonly used to treat high blood pressure (hypertension), heart failure, and diabetes Type II [3]. This compound is the prototype of antihypertensives, being formulated in tablets as COZAAR and in combination with hydrochlorothiazide as HYZAAR.

**FIGURE 2:**
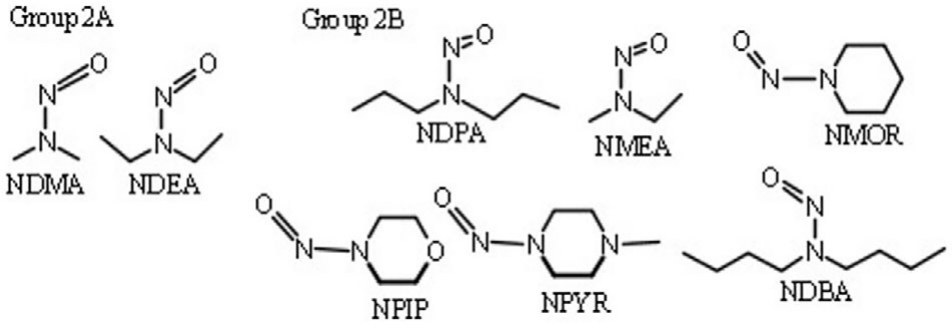
Chemical structures of nitrosamines classified as Group 2A and Group 2B.

Nitrosamines (NAs) have shown genotoxic, mutagenic, and carcinogenic effects in various animal species, according to the World Health Organization and International Agency for Research on Cancer. Thus, the WHO/IARC classifies NDMA and NDEA as Group 2A—probably carcinogenic to humans [4]. Other NAs —NDPA, NMEA, NMOR, NPYR, NPIP, and NDBA—are classified as Group 2B—possibly carcinogenic to humans (Figure 2).

In July 2018, the Chinese supplier Zhejiang Huahai Pharmaceuticals Co. regarding the potential contamination of NAs in ARB medicines, particularly in valsartan products, which was reported to have concerning levels of N‐nitrosodimethylamine (NDMA) [5]. Based on this, European Medicines Agency (EMA), United States Food and Drug Administration (USFDA), and Health Canada, among other regulatory agencies, have issued recalls regarding valsartan and valsartan‐containing products. After a deep investigation, other active pharmaceutical ingredients (APIs) including ranitidine, nizatidine, losartan, irbesartan, and metformin were also under investigation, suspected to contain NAs impurities, which also led to the recall of batches [6, 7, 8].

The presence of impurities in pharmaceuticals and medicines is a global risk that all products are subjected, in special in the current days where the production and consumption are globally elevated. Analytical aspects addressed to methodology and stability studies are crucial to ensure product safety, effectiveness, and stability throughout its shelf life, contributing to drug therapy safety by minimizing adverse effects of impurity/degradation products (DPs) [9]. In addition, the stability studies are developed to assess the quality of the pharmaceutical under influence of environmental factors like temperature, humidity, light, and considering the intrinsic product properties, including API and excipients, pharmaceutical form, and manufacturing processes, complying with the legal requirements and quality attributes within required parameters [10, 11]. The International Conference on Harmonisation (ICH) guidelines more clearly described the establishment of a stability‐indicating method, with official documents such as ICH Q1A (R2) guideline “Stability Testing of New Drug Substances and Products”, formalizing the importance of stability studies to ensure the stability of new drug substances and products [10], impurities in drug substances [11], impurities in drug products [12], evaluation of stability data [13] and potential carcinogenic impurities in drug substances and drug products (ICH) [14].

Recently additional tools have been employed for better understanding about drug stability, DPs and impurities. In a complementary field to toxicology, a modern tool and accepted as predictive is the in silico model, traditionally used for medicinal chemistry and drug design [15, 16]. Computational chemistry and in silico drug design have many approaches, including quantitative structure–activity relationships (QSAR), virtual screening, protein structure modeling, pharmacophore modeling, structure‐based drug design, and the modeling of absorption and metabolism [17]. In the specific case of NAs and eventual influence on stability profile, the in silico assessment provides valuable insights and mechanisms behind understanding of possible chemical degradation pathways under specified reaction conditions such as temperature, pH, water, oxygen, oxidation, light, metal ions, and radical initiators. These conditions are typically employed in forced degradation studies. In specific to losartan, some stability studies can be mentioned, since are important references that mention about losartan susceptibility and main DPs under stress conditions, in special photolysis and acid hydrolysis [18, 19, 20, 21]. In addition, excipients and their known impurities can be compared to a drug substance to assess the reactivity risk between them using an integrated excipient database [22].

Zeneth Nexus (version 9) and Spartan 08 (version 116.2TM; Wavefunction, Inc., USA) software are usually employed for in silico assessment addressed to DPs, potential decomposition pathways and possibly reactivity of NAs [23]. Zeneth Nexus is an advanced program designed for in silico prediction with recognized application to forced degradation and DPs of APIs under various environmental variables using a scientific literature‐based database [24, 25]. Spartan 08 is a computational‐assisted tool based on quantum mechanical calculations directly derived from the physical‐chemistry principles that shape the molecular structure. Among various chemical properties reported by Spartan 08, the energies of the frontier electron densities (FEDs) of the lowest unoccupied molecular orbital (*E*_LUMO_) and the highest occupied molecular orbital (*E*_HOMO_) are key in predicting or measuring the reactivity of impurities (NAs) in the presence of losartan under stress conditions [26]. Furthermore, the molecular electrostatic potential (MEP) map is an effective tool for understanding the impact of electronic effects and molecular fields on the molecular behavior of compounds [27, 28, 29].

In the present study the stability profile of losartan under influence of NAs impurities was investigated. For this, stability study protocols were performed testing different stressing conditions, followed by analysis using well‐established techniques like HPLC and LC‐MS associated with computational methods like Zeneth Nexus and Spartan 08. The reactivity parameters related to NAs and losartan were critically evaluated during in silico experiments and helped the interpretation of quantitative results obtained from degraded sample analysis.

## 2 Material and Methods

### 2.1 Chemical and Reagents

The raw material for the API losartan (98.75%) (drug substance), free from NAs, was purchased from IPCA Laboratories Ltd, (Mumbai, India.) The NAs reference substances, with a purity of 99.0%, included NDMA, N‐nitrosodiisopropylamine (NDIPA), and N‐nitrosodibutylamine (NDBA), which were purchased from Sigma‐Aldrich (St. Louis, Missouri, USA) at a concentration of 5 mg mL^−1^. N‐Nitrosodiethylamine (NDEA) was acquired from Toronto Research Chemicals (TRC) at the same concentration of 5 mg mL^−1^ (Toronto, Ontario, Canada). Phosphoric acid was purchased from Merck (Darmstadt, Germany). Acetonitrile HPLC (High Performace Liquid Chromatography) grade was obtained from J.T. BAKER, 99.9%. The ultrapure water was obtained from a Milli‐Q apparatus (Millipore, Milford, MA, USA) was used to prepare the sample, standard solutions and mobile phase.

### 2.2 Standard and Sample Solution Preparations

#### 2.2.1 Preparation of NAs Reference Substances Solution

To prepare the stock solutions, four NAs reference substances (NDMA, NDEA, NDIPA, and NDBA) were individually dissolved in methanol to achieve a concentration of 1 mg mL^−1^. In this study losartan samples were spiked with each NA individually, avoiding a combined mixture of impurities. This procedure is important to control eventual variability from matrix complexity. So, spiking samples containing each NA individually were prepared by adding an aliquot corresponding to 100 µg mL^−1^ or 100 (ppm) of each impurity to losartan samples at concentration 4 mg mL^−1^ in a 10 mL volumetric flask. An additional dilution was done to obtain the final concentration of losartan for analysis that corresponds to 80 µg mL^−1^. For comparison, samples of losartan API absent of NAs were also prepared in the same conditions of concentration and volume.

#### 2.2.2 Stock and Working Solutions of losartan Potassium Drug Substance

Stock solution of drug substance (API) with a concentration of 8 mg mL^−1^ was prepared by accurately weighing approximately 800 mg of losartan potassium (with a purity of 98.75%) and placing it into a 100 mL volumetric flask containing Milli‐Q water. The final solution was sonicated for 20 min. The flask was then filled with Milli‐Q water to achieve the desired volume. Working solutions were prepared just before use by diluting the stock solution to appropriate concentration levels, again using Milli‐Q water as the diluent. All the samples were filtered using a 0.45 µm filter and a 0.22 µm filter prior to injection into HPLC and LC‐MS, respectively.

### 2.3 Stability of losartan Under Influence of NAs

The samples were submitted to stress conditions to obtain a stability profile. Stock solutions of losartan containing individually each NA (100 ppm) or not were submitted to acid, basic, thermal, oxidative, and photolytic degradation, as follows:

(a) *Acid Hydrolysis*: This test was performed in a 10 mL volumetric flask. Initially, 5.0 mL of a stock solution of losartan at a concentration of 8 mg mL^−1^ was added to the equivalent of 100 ppm (µg mL^−1^) of NAs reference substances (initially concentrated at 1 mg mL^−1^) and 2.0 mL of 5.0 mol L^−1^ HCl. The flask was completed with water and stored for 12 h kept protected from light at room temperature (25°C), with the stressing agent at a final concentration of 1 mol L^−1^ HCl. After the time, the solution was neutralized by dilution adding a calculated volume of NaOH equivalent to 1.0 mol L^−1^. The neutralized mixture was then diluted with Milli‐Q water to achieve a final concentration of 80.0 µg mL^−1^ of losartan and 1 ppm of NA. The same procedure described above was performed for samples not intentionally added of NAs.

(b) *Basic Hydrolysis*: This test was performed in a 10 mL volumetric flask. Initially, 5.0 mL of a stock solution of losartan at a concentration of 8 mg mL^−1^ was added to the equivalent of 100 ppm (µg mL^−1^) of NAs reference substances (initially concentrated at 1 mg mL^−1^) and 2.0 mL of 5.0 mol L^−1^ NaOH. The flask was completed with water and stored for 12 h kept protected from light at room temperature (25°C), with the stressing agent at a final concentration of 1 mol L^−1^ NaOH. After the time, the solution was neutralized by dilution adding a calculated volume of HCl equivalent to 1.0 mol L^−1^. The neutralized mixture was then diluted with Milli‐Q water to achieve a final concentration of 80.0 µg mL^−1^ of losartan and 1 ppm of NA. The same procedure described above was performed for samples not intentionally added of NAs.

(c) *Thermal Degradation*: This test was performed in an amber flask. Initially, 5.0 mL of a stock solution of losartan at a concentration of 8 mg mL^−1^ was added to the equivalent of 100 ppm (µg mL^−1^) of NAs reference substances (initially concentrated at 1 mg mL^−1^). Thermodegradation studies were conducted in a temperature‐controlled oven at 60°C for 12 h. After the time, the mixture was transferred to a 10 mL volumetric flask and completed with Milli‐Q water. Finally, this solution was diluted with Milli‐Q water to achieve a final concentration of 80.0 µg mL^−1^ of losartan and 1 ppm of NA. The same procedure described above was performed for samples not intentionally added of NAs.

(d) *Oxidative Degradation*: This degradation assay was performed in a 10 mL volumetric flask. Initially, 5.0 mL of a stock solution of losartan at concentration of 8 mg mL^−1^ was added to the equivalent of 100 ppm (µg mL^−1^) of NAs reference substances (initially concentrated at 1 mg mL^−1^) and 0.5 mL of 30% H_2_O_2_, being completed with Milli‐Q water. The final solution containing of 3% H_2_O_2_ was stored kept protected from light at room temperature (25°C) for 12 h. After the time, this solution was diluted with Milli‐Q water to achieve a final concentration of 80.0 µg mL^−1^ of losartan and 1 ppm of NA. The same procedure described above was performed for samples not intentionally added of NAs.

(e) *Photolytic Degradation*: This test was conducted by adding 1 mL of a stock solution of losartan 8 mg mL^−1^ to an equivalent of 100 ppm (µg mL^−1^) of NAs reference substances (initially concentrated at 1 mg mL^−1^) in Plastibrand disposable cuvettes. The photodegradation studies were carried out in a photostability UV chamber (1.0 m × 0.17 m × 0.17 m) equipped with UV‐A lamp Ecolume ZW 352 nm 30 W. The samples were then subjected to UVA radiation at room temperature (25°C) for 12 h. After exposure, they were diluted with Milli‐Q water to achieve a final concentration of 80.0 µg mL^−1^ of losartan and 1 ppm of NA. The same procedure described above was performed for samples not intentionally added of NAs.

### 2.4 Instrumentation and Conditions

#### 2.4.1 HPLC

The chromatographic analysis was conducted on a Shimadzu HPLC modular system (Shimadzu, Kyoto, Japan) equipped with System Controller (CBM‐20A), Solvent Delivery Unit (LC‐20AD), On‐line Degassing Unit (DGU‐20A), and Photo‐diode Array (PDA) detector (SPD‐M20A). Peak area integration was performed automatically by Shimadzu LC Solution V1.24 SP1. The samples were introduced into the Auto‐Sampler (SIL), which featured a dual solvent delivery system. The chromatographic separation was performed on a PhenomenexRP‐18 column (150 mm × 4.6 mm, 5 µm). The elution was carried out at a flow rate of 1.0 mL min^−1^ using an isocratic system formed by a mixture of mobile phase A (purified water adjusted to a pH of 2.5 using 10% (v/v) o‐phosphoric acid) and mobile phase B (mixture of 90% purified water and acetonitrile) in a 55:45 ratio. Before using, the mobile phase was filtered through a 0.45 µm filter and sonicated for 15 min. The injection volume was 20 µL, and the run time was set to 8 min. The column oven temperature was maintained at 25°C, and losartan was detected using UV photodiode array detection at a wavelength of 254 nm (*Brazilian Pharmacopeia ‐Brazilian National Formulary‐V [sixth edition]*) National Health Surveillance Agency [30].

#### 2.4.2 LC‐MS

The LC‐MS method was performed using a Nexera UFLC (Shimadzu, Kyoto, Japan) equipped with two binary pumps (LC‐30AD), a column oven (CTO‐30A), a diode array absorbance detector (SPD‐M20A), and an automatic injector (SIL‐30AMP) coupled to a triple quadrupole mass spectrometer model LCMS‐8045 (Shimadzu, Kyoto, Japan). The chromatographic separation of NAs was carried out using a Purospher STAR RP18 column (2.1 mm × 50 mm × 2.0 µm) from Merck following an elution by gradient. For this, a solvent gradient program of 0.1% formic acid in ultrapure water as mobile phase A and MeOH as mobile phase B was implemented at a flow rate of 0.25 mL min^−1^. The initial gradient of 5% B was held for 1.0 min. The gradient was increased linearly to 50% B within 6.0 min keeping until 6.50 min, increased to 100% B up to 12.0 min, after returned to 5% B and where it remained stable until the end of the run (20 min). The temperatures of analytical column and autosampler were kept at 40°C and 5°C, respectively [21].

MS detection was performed applying the atmospheric pressure chemical ionization (APCI) source in positive ion mode. The following conditions were applied: heat block temperature was set to 200°C; nebulizer gas (N_2_) flow was adjusted to 3.0 L min^−1^; desolvation line temperature was maintained at 602°C; drying gas (N_2_) flow was set to 5.0 L min^−1^, and the collision‐induced dissociation gas pressure (Ar) was maintained at 230 kPa. The analyses were conducted in multiple reaction monitoring (MRM) mode. To enhance sensitivity and specificity, additional detection parameters were optimized, including Q1 Pre Bias (voltage to promote the ionization of the precursor ion), Q3 Pre Bias (voltage to promote the ionization of the product ion), and collision energy. For each analyte, three MRM transitions were selected: one for quantification and two for qualification, as follows NDEA *m*/*z* 103.20 > 29.00, *m*/*z* 103.20 > 75.15, *m*/*z* 103.20 > 27.15; NDIPA *m*/*z* 131.20 > 43.15, *m*/*z* 131.20 > 89.15, *m*/*z* 131.20 > 41.05; NDBA *m*/*z* 195.20 > 57.20, *m*/*z* 195.20 > 41.05, *m*/*z* 195.20 > 103.10. Data extraction was performed using LabSolutions software (Shimadzu, Kyoto, Japan). A full scan analysis with a mass‐to‐charge ratio (*m*/*z*) range of 10–500 was also conducted to verify whether NAs and DPs were formed under the degradation conditions tested.

### 2.5 Computational Chemistry Studies

#### 2.5.1 Zeneth Software

The Zeneth Nexus software (version 9.0.1) for Windows (Lhasa Limited, Leeds, UK) is a predictive degradation expert software designed to identify plausible DPs of drug compounds. This commercial software program uses information‐based transformations to accurately predict the breakdown pathways of specific molecules based on their structures. It uses extensive database knowledge based on degradation mechanisms to quickly predict the formation of expected DPs available in the literature [31].

This program uses information‐based transformations to accurately predict the breakdown pathways of specific molecules based on their structures. The software is specifically tailored to predict chemical degradation pathways in silico for pharmaceuticals (drug substances and drug products). However, the Zeneth software may generate more DPs than those commonly observed in practice. The software functionality depends on several factors, including the inter‐chemical structure, reaction conditions, maximum number of steps allowed, and the probability threshold associated with the selected query molecule [32]. For predictions, five sets of stress conditions are provided: acidic hydrolysis, basic hydrolysis, thermal degradation, photodegradation, and oxidation (H_2_O_2_).

Here in the present study the DPs will be predicted and an associated likelihood score will be assigned to each functional group from the target drug that corresponds to the degradation reaction studied. The likelihood score of a DP attempts to estimate the likelihood of the degradation reaction occurring. Likelihood scores is a probability‐based measure ranging from 0 to 1000, with 0 being impossible, any score greater than 599 being likely, and 1000 being considered certain to occur [24]. Zeneth does not consider concentration or reaction time, nor performs calculations such as reaction kinetics or transition state energies. Obtaining appropriate kinetics data and sufficient information on the substrate dependence of reactivity can be difficult. Although reactivity data can be found in the synthesis literature, Zeneth likelihood scoring is designed to be relevant to forced degradation studies. These studies typically occur at room temperature or slightly higher, rarely exceeding 80°C, and with time scales from minutes to weeks. In addition, they generally aim for 5%−20% degradation of the DS [33].

The primary data inserted on Zeneth is the structure of the query compound. Furthermore, parameters known as processing constraints are used to control prediction. Among some limitations, response conditions, the number of reaction steps (pathway length), the minimum probability level, the inclusion or exclusion of dimerization reactions, and whether or not tautomerization of reacting compounds is considered. While following the guidelines and recognizing all relevant transformations from literature or previous experimental data (knowledge base), the program creates the first level or step of DPs. Each predicted DP will be processed similarly like the drug substance if more than one step is permitted. This process will be repeated until the user‐specified number of steps has been completed [34].

The theoretical knowledge base content is derived from various sources, including books on chemical degradation [35, 36, 37], general chemistry textbooks [38] and primary literature, as well as proprietary data obtained through ongoing data‐sharing initiatives with Zeneth users. The knowledge base used in this study includes 531 transformation types and comprises six categories of degradation reactions: hydrolysis (124), oxidation (154), photodegradation (43), isomerization (34), elimination (64), and condensation (112) The knowledge database is constantly refined and expanded as new knowledge is gathered from these sources.

Furthermore, the process is informed by the expertise of scientists at Lhasa Limited, as well as data‐sharing contributions from Zeneth user community. The prevalence of scores such as 500, 700, and 900 in the predictions reflects their alignment with the nonnumerical likelihood classifications—“equivocal,” “likely,” and “very likely”—that have been consistently utilized in all prior versions of the software.

A step score is determined by evaluating an individual transformation type and is only dependent on the specified conditions and structural features of the query compound that affect the chemical reactivity. The pathway likelihood calculation scores of a degradation pathway (“pathway score”) are calculated from the scores of the individual steps that lead to the DP. There are two ways to calculate a pathway score based on step scores. The first and simplest is the Lowest Step Likelihood (LSL) approach, which is the software's default setting and sets the pathway score to the least likely step's score. This method was applied in this work. The second is Multiplied Step Likelihood (MSL), in which the pathway score is determined by multiplying the product of these probabilities by 1000 and interpreting the step scores as probabilities (i.e., divided by 1000). Although the LSL approach is simple, it offers little granularity for longer pathways. For instance, a four‐step pathway (B) with 700 scores for each step produces a 700 pathway score; a different four‐step pathway (A) with 700 scores for the first step and 900 (very likely) for the subsequent steps also produces a 700 pathway score. However, Pathway B, a concatenation of likely steps, seems less likely than Pathway A [24].

The method used in the Zeneth software predicts DPs that have been experimentally observed and/or identifies types of degradation commonly seen in the literature, applying a score of at least 500 (equivocal). Ester hydrolysis, for example, are typically predicted with a score of 700 (likely) around pH 7, and 900 (very likely) at any other pH. The assignment of these scores is contingent upon the quality and quantity of available literature, which encompasses data on degradation pathways, physicochemical properties, and synthetic routes. In addition, the expertise of scientists at Lhasa Limited, along with data‐sharing contributions from Zeneth's users, play a crucial role in this process. Scores of 500, 700, and 900 are highly prevalent in predictions because they are based on the nonnumerical likelihood assignments of “equivocal,” “likely,” and “very likely,” in all previous versions of the software [39, 40].

#### 2.5.2 Spartan Software

Spartan 08 version 116.2TM (Wavefunction, Inc., USA) was used to conduct the computational analyses, with all initial structures obtained using the software default settings and structural fragments from its molecular editor. The Spartan dataset served as the basis set for optimizing the compounds under assessment and a series of descriptors were calculated for further analysis. The global reactivity descriptors for NAs including chemical potential (*μ*), chemical hardness (*η*) chemical softness (*S*), electron affinity (EA), ionization energy (IE) electrophilicity (*ω*) nucleophilicity (*N*) polarizability (*α*) HOMO–LUMO gap (Δ*E*_HOMO–LUMO_ **),** LUMO energy (*E*_LUMO_) HOMO energy (*E*_HOMO_), Δ*E*_back‐donation_ maximum charge transfer (Δ*N*_max_) were calculated using density functional theory (DFT). Spartan 8 software is a computational‐assisted tool based on quantum mechanical calculations that are directly derived from the physical‐chemistry principles and properties that govern the molecular structure approach by solution of the Schrödinger equation in an approximate way.

DFT to determine chemical functions is widely used in generating models which enable understanding and predicting the structure degradation of drugs [41]. MEP, highest occupied molecular orbital—*E*_HOMO_ ‐, *E*_LUMO_ ‐, are used to study alkaline or acid hydrolysis, autooxidation and photolytic reactions [26], respectively. Moreover, the theoretical quantum chemical calculations for the derivatives were performed at the DFT using Spartan‐8 software to optimize molecular geometry, and several properties of all the series of compounds were computed [42].

The MAPs of the four NAs discussed in this study were build using Spartan ’10. Spartan ’10 calculates the electrostatic potential at selected points on the 0.002 isodensity surface and maps the surface by color, where different colors are used to identify different potentials. The electrostatic potential varies from most negative (red) to most positive (blue) and less negative (red) as follows: red < orange < yellow < green < blue. For this, quantum mechanical methods can be employed depending on the required accuracy such as Hartree–Fock (HF), DFT, or semi‐empirical methods. Geometry optimization was conducted in three steps. First, the Merck Molecular Force Field (MMFF94) was applied. Next, a new optimization was performed using the Austin Model (AM1). In the final stage, re‐optimization was carried out using DFT at the B3LYP/6‐31G* (d, f) basis set level of theory. In this stage, the number of electrons in a natural population analysis (NPA) was calculated using the single‐point energy at the same level of theory as the geometry optimization [43].

## 3 Results and Discussion

In recent years, the discovery of potentially carcinogenic contaminants in ARBs has received special attention from scientists, regulatory body and population. Quality control analysis become very important as preventive action due the risks involved with that substances. In the context of stability studies, decisive for approval of API and drug products, the presence of NAs may be critical in terms of DPs, influence on this generation, degradation rate and even possibly for formation of nitroso‐drug derivatives. Also, the consumption of NAs or even their in situ generation eventually observed in the samples can indicate a real need for deep investigations. Furthermore, information about eventual influence on shelf life are important for marketing authorization holders. This need is further emphasized by the lack of conclusive data in the literature regarding this theme here raised.

### 3.1 Stability of Losartan Versus NAs—HPLC Analysis

To conduct an experimental protocol according to the legal requirement it is important to follow international references and guidance. Here, the degradation tests followed the recommendation harmonized by ICH [13, 44] for forced degradation studies by hydrolysis (acid and basic), oxidation, thermal, and photolysis.

**FIGURE 3:**
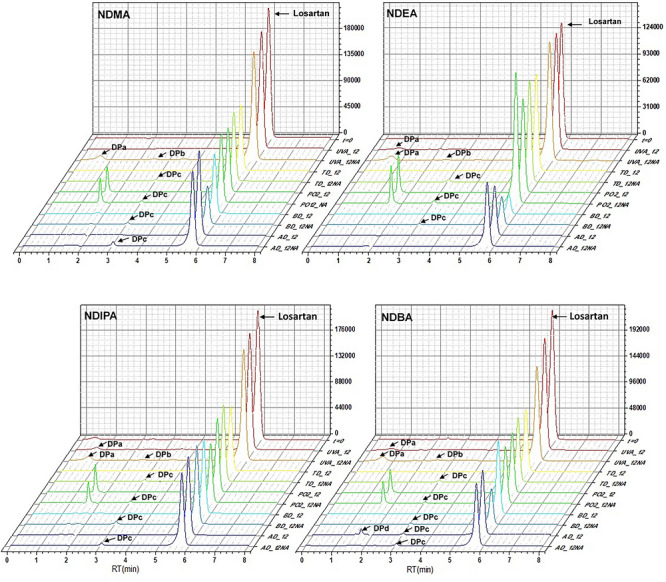
Representative HPLC chromatograms of losartan and its degradation products from stability study testing different degradation conditions in samples containing or not nitrosamines (NAs)—NDMA, NDEA, NDIPA, and NDBA. UVA: UVA photolysis; TD: Thermal degradation; PO: peroxidation; BD: basic degradation; AD: acid degradation; NA: presence of nitrosamine; 12:12 h time of degradation; DPa, DPb, DPc, DPd: degradation products detected during HPLC analysis; retention time of losartan: 5.7 min.

For assay, losartan API samples were prepared considering the intentional addition of four NAs (NDMA, NDEA, NDIPA, and NDBA) at final concentration of 100 ppm (µg mL^−1^) prior to the corresponding forced degradation process. For comparison, samples free of NAs were also studied. The results from HPLC‐DAD analysis (Figure 3) indicated the presence of some DPs in the chromatographic run, sometimes repeated depending on the NA added, as follows: photolysis (UVA_12NA) (DPa and DPb,) thermal degradation (TD_12NA) (DPc), oxidative degradation (PO2_12NA) (DPc), basic hydrolysis (BD_12NA) (DPc), and acidic hydrolysis (AD_12NA) (DPc). The addition of NDBA in the samples subjected to acidic hydrolysis (AD_12NA) resulted in a DPd DP not observed for other NAs. Observing the results from samples absent of NAs, the degradation by photolysis deserves more attention with the compound DPa (UVA_12), being detected for all stressing conditions.

Based on this context described above, the presence of NAs significantly affected the number of DPs in all degradation tests. Notably, the highest number of DPs—seven in total—was generated when NAs were present, being observed a considerable number from UVA photolysis (three DPs), even considering an elevated residual drug concentration, approximately 90%. Despite of some few differences, a comparative general overview of the chromatograms reveals a similar profile between samples with NAs and absent of them. Table 1 shows the quantitative data obtained from this stability testing, revealing losartan residual concentration from each degradant factor and NA tested.

**TABLE 1:** Losartan residual content (%) obtained from quantitative determination in degraded samples added or absent of nitrosamines (NAs) (NDMA, NDEA, NDIPA, and NDBA). Analytical determination by HPLC.

NDMA	(Los) (%) ± SD (absence of NAs—100 ppm)	RSD (%)	(Los) (%) ± SD (presence of NAs—100 ppm)	RSD (%)	*t*‐tab	*t*‐cal	*p*‐value	Significance^a^
Acid hydrolysis	49.52 ± 2.15	4.34	41.87 ± 0.86	2.68	2.13	5.71	0.002	S
Basic hydrolysis	44.02 ± 4.50	9.98	28.88 ± 3.48	10.55	2.13	5.17	0.003	S
Thermal degradation	41.89 ± 1.00	2.39	42.55 ± 0.72	1.70	2.13	0.92	0.203	NS
Oxidation	42.12 ± 0.78	1.80	44.52 ± 0.79	1.80	2.13	3.52	0.012	S
Photolysis	91.09 ± 1.95	2.14	87.64 ± 1.35	1.35	2.13	4.03	0.024	S
								
**NDEA**	**(Los) (%) ± SD (absence of NAs—100 ppm)**	**RSD (%)**	**(Los) (%) ± SD (presence of NAs—100 ppm)**	**RSD (%)**	***t*‐tab**	***t*‐cal**	***p*‐value**	**Significance** ^a^
Acid hydrolysis	35.09 ± 3.82	8.28	46.03 ± 5.53	5.53	2.13	3.77	0.002	S
Basic hydrolysis	22.43 ± 1.78	10.06	28.64 ± 2.03	7.09	2.13	3.97	0.008	S
Thermal degradation	49.06 ± 1.06	1.26	49.79 ± 0.63	1.26	2.13	1.03	0.179	NS
Oxidation	46.92 ± 0.30	0.65	43.23 ± 2.02	4.44	2.13	3.34	0.014	S
Photolysis	95.98 ± 2.54	2.65	88.88 ± 5.19	4.85	2.13	3.57	0.011	S
								
**NDIPA**	**(Los) (%) ± SD (absence of NAs—100 ppm)**	**RSD (%)**	**(Los) (%) ± SD (presence of NAs—100 ppm)**	**RSD (%)**	***t*‐tab**	***t*‐cal**	***p*‐value**	**Significance** ^a^
Acid hydrolysis	50.87 ± 0.65	1.29	52.86 ± 0.29	1.11	2.31	4.56	0.008	S
Basic hydrolysis	52.49 ± 9.08	17.29	51.19 ± 3.70	7.23	2.13	2.17	0.037	S
Thermal degradation	45.49 ± 3.39	7.45	53.06 ± 2.93	5.74	2.13	1.62	0.123	NS
Oxidation	50.60 ± 0.60	1.28	43.87 ± 0.50	1.14	2.13	14.20	0.001	S
Photolysis	94.92 ± 2.28	2.24	89.06 ± 1.19	1.33	2.13	4.25	0.012	S
								
**NDBA**	**(Los) (%) ± SD (absence of NAs—100 ppm)**	**RSD (%)**	**(Los) (%) ± SD (presence of NAs—100 ppm)**	**RSD (%)**	***t*‐tab**	***t*‐cal**	***p*‐value**	**Significance** ^a^
Acid hydrolysis	44.81 ± 2.16	4.83	48.86 ± 0.12	0.24	2.13	3.19	0.046	S
Basic hydrolysis	34.99 ± 4.81	13.75	33.34 ± 3.96	1.88	2.13	2.46	0.038	S
Thermal degradation	45.80 ± 1.86	4.07	47.70 ± 1.23	2.59	2.13	1.32	0.128	NS
Oxidation	55.63 ± 5.74	5.31	44.79 ± 2.86	6.39	2.13	2.92	0.021	S
Photolysis	94.24 ± 2.45	2.60	80.20 ± 1.52	0.24	2.13	10.14	0.004	S

Abbreviation: Losartan: LOS.

^a^ Significance (S) and nonsignificance (NS) determined by *t*‐test (5% of significance).

Observing these quantitative values, the individual measuring of residual content of losartan helped to clarify initially the effect of NAs on each forced degradation processes. In general, losartan residual contents are lower in the samples containing NAs for oxidation and photolysis, except for oxidation when NDMA is present. For acid and basic hydrolysis, the results do not follow a well‐established pattern, being necessary an individual interpretation for getting an adequate conclusion about the influence of each NA. For both degradants (acid and basic), the samples containing NDIPA presented a residual concentration of losartan in approximately 50%, similar to degraded sample absent of this NA. In addition, the higher rate of degradation was observed for basic media, with values of 22% and 28% of drug residual content for samples added of NDEA or not, respectively. In the case of NDMA and NDBA, the basic media caused a higher degradation rate being more significant in the samples containing NAs, 28% and 33%, respectively. Observing the results from photolysis the residual content of losartan ranged 80%–90% for all conditions, even though the degradation was clearly less aggressive despite having formed more DPs as illustrated in Figure 3. Regarding the thermal degradation the drug residual concentration was not considered statistically significant in the comparison performed, that is, the presence or absence of NA had the same effect. Despite of this, this stressing factor caused a very expressive drug decomposition. Now, it is important to highlight the natural variability expected for degraded samples, which is dependent of several intrinsic and extrinsic factors that can affect the sample as a whole, being commonly uncontrollable. Also, the natural reactivity of NAs that is chemically known could not be considered due the complexity associated with these results.

### 3.2 Stability of Losartan Versus NAs—LC‐MS Analysis

**FIGURE 4:**
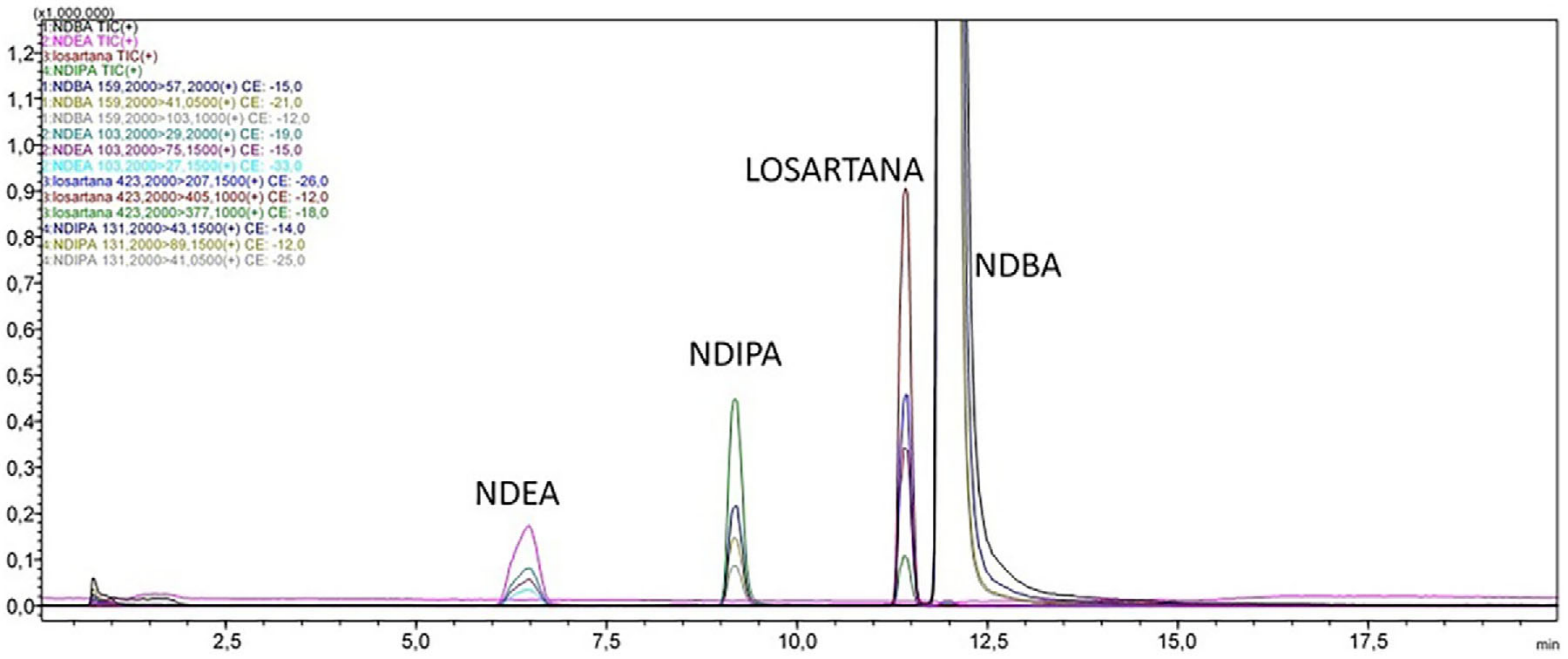
Illustrative LC‐MS chromatograms obtained from analysis of losartan active pharmaceutical ingredient. Multiple reaction monitoring transitions applying *m*/*z* values for losartan, NDEA, NDIPA, and NDBA. Samples not subjected to degradation.

Initially it is important to mention the fragmentation pattern are sensitive to specific parameters (collision energy, source conditions) for each instrument, that is, the analytical response from LC‐MS analysis using different MS platforms can suffer variability depending on the instrument used. Thus, some variability in the results is something acceptable, mainly when the complexity of samples is widely recognized. The LC‐MS analyses of losartan API samples subjected to forced degradation for 12 h in the presence or absence of NAs were conducted in APCI‐positive mode. As initial control, each NA (NDEA, NDIPA, and NDBA) added to losartan API was monitored by the quantitative LC‐MS method previously validated by our group [21] (Figure 4). The monitoring of formation or consumption of NAs was performed through specific MRM transitions: NDEA *m*/*z* 103.20 > 29.00, *m*/*z* 103.20 >75.15, *m*/*z* 103.20 > 27.15; NDIPA *m*/*z* 131.20 > 43.15, *m*/*z* 131.20 > 89.15, *m*/*z* 131.20 > 41.05; NDBA *m*/*z* 195.20 > 57.20, *m*/*z* 195.20 > 41.05, *m*/*z* 195.20 > 103.10. NDMA was not assayed in this experimental step considering that our validated LC‐MS method did not include this NA. Complementary to MRM acquisition, full scan analyses were performed and allowed acquire additional information about any different signals that could be related to eventual DPs formed. Obviously that *m*/*z* signal from known DPs were set in order to investigate its presence on degraded samples. Also, considering the needing for more reliable results from mass spectra obtained from degraded samples, the MS analytical data were processed using an elemental composition calculator, by which the most probable molecular formula relative to ion products and their mass losses were estimated.

**FIGURE 5:**
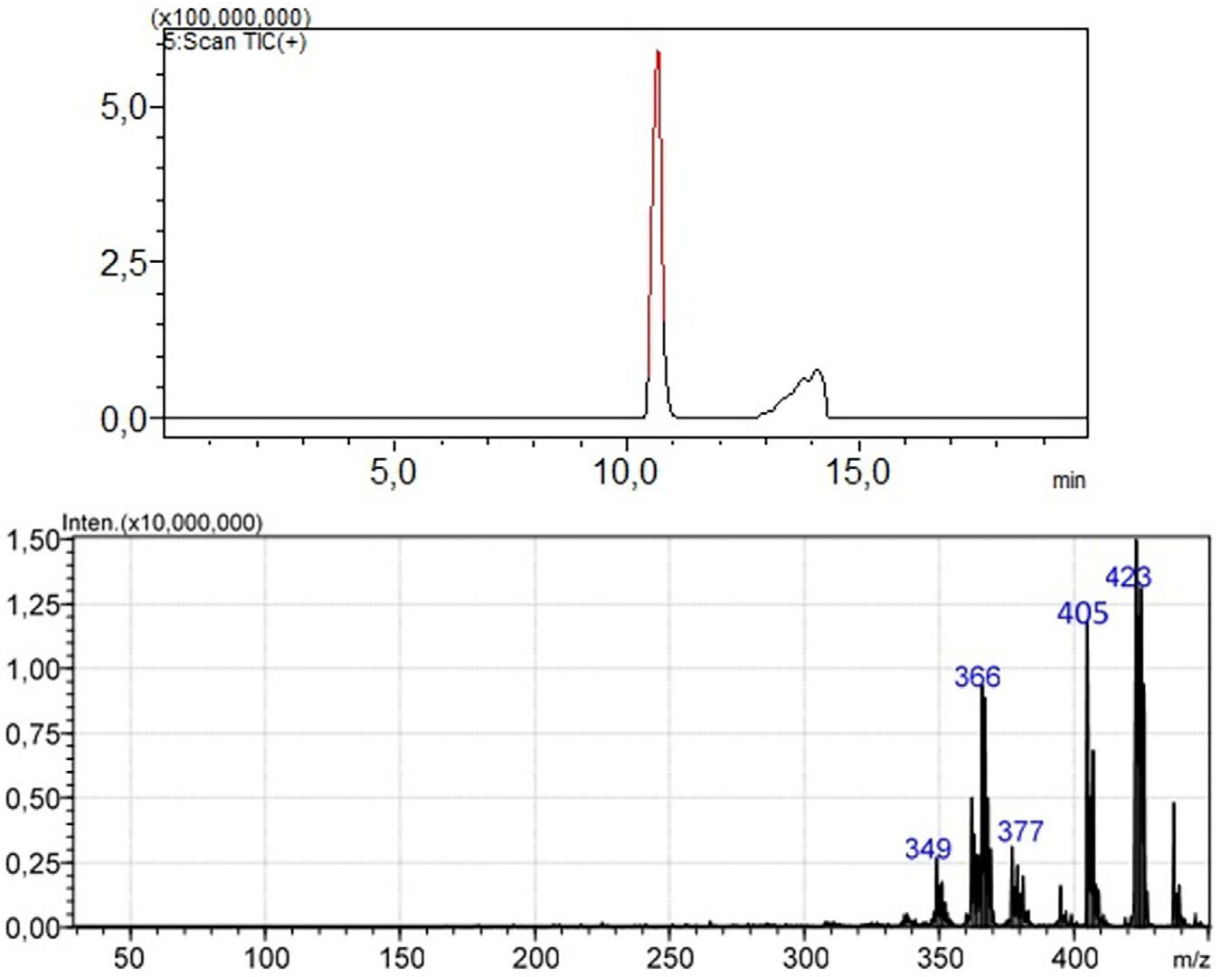
Representative chromatogram (TIC: total ion chromatogram) and full scan mass spectra of losartan active pharmaceutical ingredient. Sample ionization by atmospheric pressure chemical ionization in positive ion mode.

Initially losartan API not degraded was analyzed by a simple chromatographic run, in order to confirm its identity and eventual absence of contaminants (Figure 5). The *m*/*z* signals at 423 (molecular ion), 405, and 377 confirmed the identity of the drug, showing a mass spectra profile in accordance to the literature [21, 45]. Prior to degradation, the API sample was also analyzed through MRM data acquisition applied to NAs NDEA, NDIPA, and NDBA in order to investigate eventual absence of interferents on degradation process, whose results were confirmatory for absence of contamination in losartan API.

#### 3.2.1 NAs in Degraded Sample of Losartan—MS Data Acquisition

In a search for more information about influence of NAs on degradation process and formation or consumption of NAs during this process, the degraded samples were analyzed by LC‐MS through MRM data acquisition focused on losartan and NAs (NDEA, NDIPA, and NDBA). This data acquisition procedure was used for a real monitoring of that substances, which could help in the interpretation of chemical behavior from the degraded matrices. Similar to HPLC‐DAD analysis, the residual concentration of losartan was determined (Table 2), allowing to observe the influence of NAs on drug degradation. Each degradation condition (acid, basic, thermal, oxidative, and photolysis) was separately evaluated, allowing a more detailed interpretation.

**TABLE 2:** Losartan residual content (%) obtained from quantitative determination in degraded samples added or absent of nitrosamines (NAs) (NDEA, NDIPA, and NDBA). Analytical determination by LC‐MS triple quadrupole.

Samples degradation factor	(Los) control		(Los) + NDEA (50 ng mL^−1^)		(Los) + NDIPA (50 ng mL^−1^)		(Los) + NDBA (50 ng mL^−1^)	
	(Los% ± SD)	RSD	(Los% ± SD)	RSD	(Los% ± SD)	RSD	(Los% ± SD)	RSD
Acidic Hydrolysis	45.08 ± 5.33	11.93	63.97 ± 3.62	5.66	45.33 ± 9.02	19.09	35.85 ± 0.08	0.22
Basic Hydrolysis	55.24 ± 5.14	9.30	52.67 ± 12.49	23.71	46.10 ± 0.52	1.13	48.68 ± 0.55	1.14
Thermaldegradation	42.52 ± 5.49	12.91	43.41 ± 8.78	20.22	44.46 ± 13.60	30.58	58.34 ± 0.53	0.93
Peroxidation	42.92 ± 3.08	7.19	55.11 ± 0.70	1.26	47.92 ± 3.03	6.32	55.80 ± 0.65	1.17
Photolysis	80.68 ± 3.68	5.29	78.18 ± 5.53	7.07	77.26 ± 1.63	2.11	75.81 ± 0.70	0.93

Abbreviation: Losartan: LOS.

In parallel with the monitoring of losartan residual concentration, NAs were also monitored to obtain any information about their concentration after the degradation process, whether consumption or formation. In order to confirm the method ability to detect NDEA, NDIPA, and NDBA, analytical controls were run and the *m*/*z* transitions were detected adequately (Figure 4). For losartan API, control samples not degraded were also injected. When degraded samples were injected, only NDEA was detected in the samples intentionally added of NA, specifically in the thermal and oxidative degradation. In all other conditions, *m*/*z* signals relative to NAs were not detected for any degraded sample, including those absent of NAs.

In terms of losartan residual concentration, the samples containing NDEA showed a higher drug concentration, being more significant in oxidative (55.11 %) than thermal degradation (43.41%). In fact, thermal stress caused a similar effect in the samples absent of NAs, with a residual content of losartan in 42.52%. Hypothetically, NDEA could present a protective effect due its reactivity with the chemical agent or stressing factor. Another hypothesis is related to the stability of that NA to the degradant agent, maybe due its intrinsic reactivity. For other all samples containing or not NAs, these substances were not detected. Many interpretations can be raised, which can be associated to reactivity, stability, or interaction NA‐losartan through a nitrosation reaction [46, 47]. A nitroso compound like nitroso losartan was not investigated in this study.

A survey of literature has shown some publications describing the nitrosation reaction involving some drugs, like oxytetracycline, duloxetine, and ethambutol [47, 48, 49]. Chemically, a conversion of NA into a nitroso‐drug, or nitrosation reaction, even in a small amount is a serious risk since can leads to NA intake that is orders of magnitude above of acceptable limits [47]. Environmental factors as pH and temperature, or presence of water, can interfere on nitrosation reaction. In fact, aqueous and acid environment can catalyze nitrosation reaction [47, 50, 51]. Also, chemical equilibrium in terms of amine lone pair (of electrons) for nucleophilic attack is considered a key factor. According to Reiss et al. [47], conditions of warm and acidic with excess of nitrite are very favorable for N‐NA formation. Complementary, biological media as found in the human body must be considered as essential to understand the in vivo reactivity. Acid conditions in the stomach can catalyze the nitrosation reaction in the presence of nitrite, as well as some enzymes present in saliva and digestive tract, mainly at neutral‐to‐basic pH [52, 53]. Similar findings have been also described recently concluding that the use of less basic amines, elevated processing temperatures, or low pH conditions in combination with significant levels of nitrite have the potential to generate levels of N‐NAs. In addition, other factors (storage, pH, particle size, peroxide content, etc.) also can affect the NA content [51, 54].

Similarly to what was observed for degraded samples analyzed by HPLC‐DAD (Table 1), a significant degradation was verified in acid, basic, thermal, and oxidative conditions, which resulted in a losartan residual concentration in the range of 42%–55%. By photolysis the degradation was not significant, resulting in quantitative values approximately 80% of losartan in all samples, independent if NAs are present or not. Even though it is not quantitatively aggressive factor, photolysis is very important to be investigated in stability studies of losartan due its chemical structure containing imidazole ring [18, 21, 55]. When NAs are considered, different degradation profiles are visualized, depending on the NA or the degradant agent. In the samples containing NDBA for example, it is assumed a greater reactivity in acid and basic media, in contrast to thermal and oxidation whose results showed lower rate of degradation. The rationale for protecting the drug or increasing reactivity of NAs is complex and involves chemical aspects related to NAs reactivity [47]. For example, acid and basic media have influence on electrons rearrange and possibly chemical interactions within a complex matrix like degraded sample. It is also important to consider the protective effect from NAs, which have greater reactivity and can consume the degradant agent with varying degrees of intensity. In general, NAs exhibit increasing reactivity based on their chemical structure. Chemically, the reactivity of alkyl NAs increases with increasing molar mass. So, NDBA is the most reactive, followed by NDIPA and NDEA.

Observing the influence of NDEA on losartan degradation, this NA showed a protective effect against losartan in acid hydrolysis. As described above, acid pH is favorable to nitrosation reaction, differently than observed here. Even considering the complexity of degraded samples, the reactivity between this NA (NDEA) and the degradant factor that results in a protective effect could be suggested. In the oxidative sample, the presence of NDEA had also a protective effect (residual losartan = 55.11%) when compared to the degraded samples absent of NAs (residual concentration of losartan = 42.92%). As already mentioned here, this effect is probably due to reactant (H_2_O_2_) consumption, in addition to the natural complexity of degradation medium that causes variability between samples. In addition, possible DPs could be generated in parallel to consumption of NAs, becoming more difficult the data interpretation around drug residual content, NAs concentration, and reactivity. It is important to consider that NAs were added on degraded samples before starting the degradation reaction, perhaps being responsible for the lack of reproducibility in the degradation reaction or becoming difficult to obtain a standardized profile in this stability study.

For degraded samples containing NDIPA, the results showed a similar profile between samples added of NAs or absent of them, with exception of basic hydrolysis, where the residual concentration of losartan (46.10%) was lower in the spiked samples. A correct interpretation about this result is not simple to make, but considering general aspects involving mainly the reactivity of substances, an interaction between losartan and NAs can indeed be raised. Chemically ionizable due to its two *p*K_a_ values, losartan ionizes depending on the medium and presence of ionizing agent. For obvious, the reactant (degradant‐NaOH) is responsible for that chemical modification on drug, but in the presence of NA there is a natural influence of this compound which possibly attack chemical groups that are feasible to access. Here it is important to mention NAs are partially stable at neutral and basic pH, but its reactivity is dependent of the medium as a whole, other components, solvent reactivity, and pH.

With regard to the reactivity of NAs, they are known to be stable at neutral and strongly basic media in the absence of light. Chemically, NAs can undergo modifications when exposed to ultraviolet light, which causes decomposition into aldehydes, nitrogen, and nitrous oxide, or amines and nitrous acid. Depending on the pH range that solutions are prepared, NAs may remain stable, as mentioned before. Neutral and alkaline medium favors stability, differently of acid solution which can be responsible for decomposition by denitrosation reaction. Denitrosation reaction is also expected to occur in the presence of nucleophiles [56].

In a real context applied to analytical routine found in the market, industry or academy, our results are contributive to prevent eventual analytical results not expected for a routine analysis based on impurities, NAs and stability. Eventually, a stability study could be performed thinking in the complexity of composition, mainly when there are risks for presence of NAs. Currently, many regulatory documents guide the industry and academy to have caution with pharmaceuticals that have tendency to contain or form NAs, being very important to consider these impurities during stability protocols.

#### 3.2.2 MS Full Scan for the Identification of DPs

**FIGURE 6:**
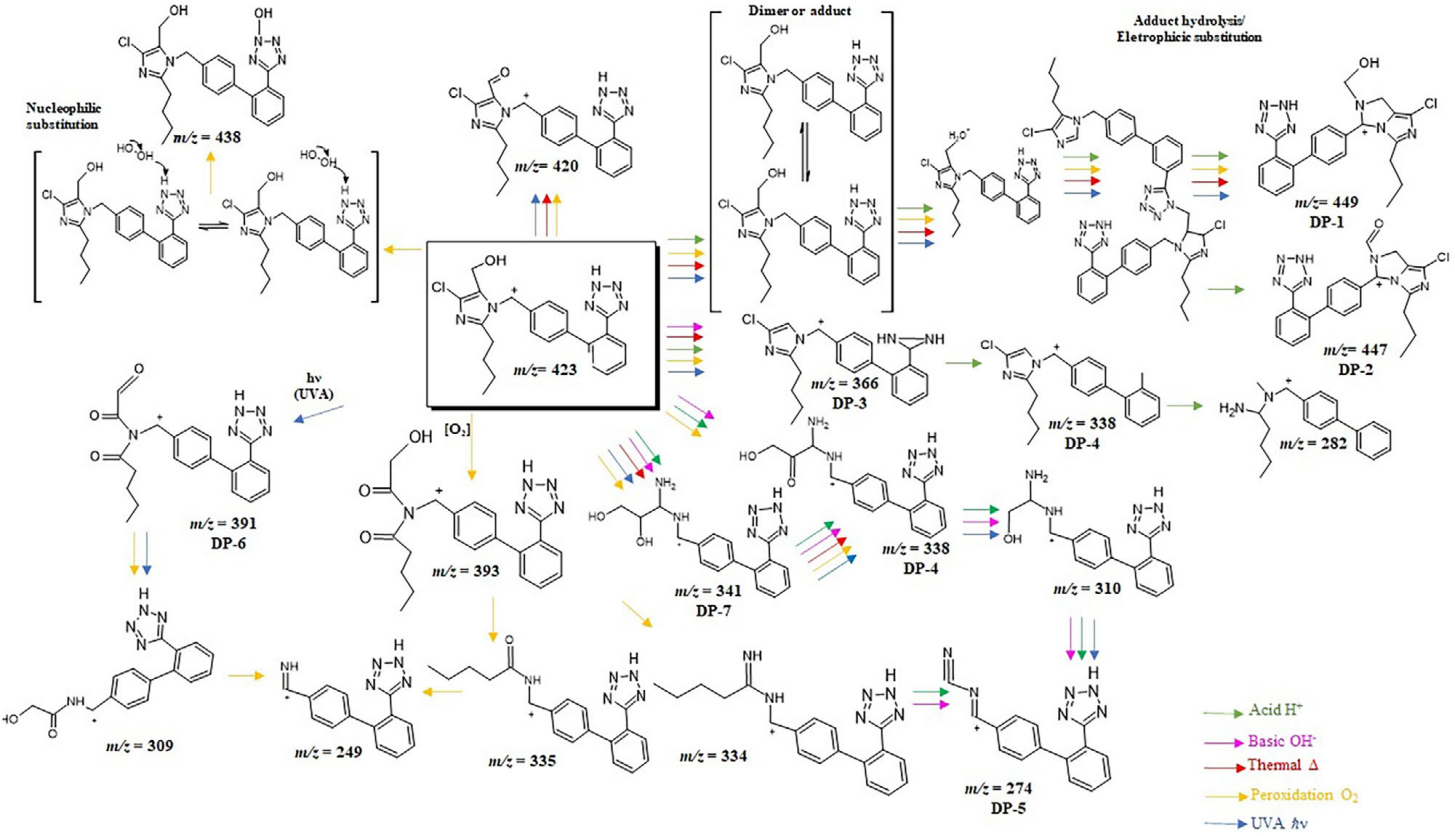
Fragmentation pathway proposed for chemical decomposition of losartan based on data obtained by LC‐MS on samples of forced degradation in the presence or absence of nitrosamines (NAs). DP: degradation product; losartan at *m*/*z* 423.

**FIGURE 7:**
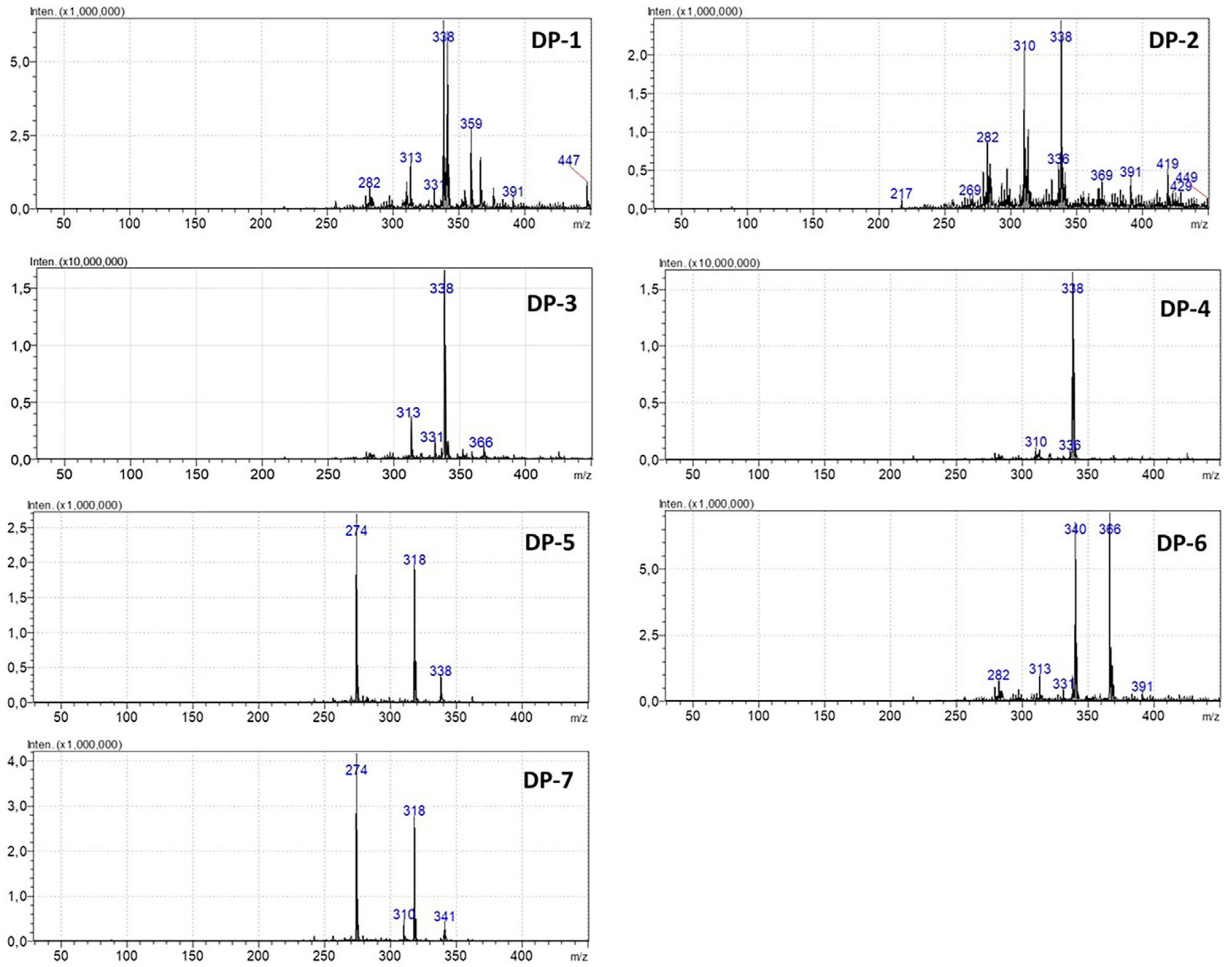
Mass spectra of degradation products obtained through triple quadrupole LC‐MS analyzes of losartan degraded samples, testing the effect of nitrosamines in the sample composition. DP: degradation product.

The degraded samples added or not of NAs were analyzed through MS full scan in parallel to MRM acquisition in order to detect DPs eventually generated under stress conditions. This strategy was used to enable chemical mapping of the DPs and possible chemical routes involved. Obviously, previous literature [18, 20, 21, 55] was used to help the correct elucidation and probable chemical modifications from decomposition under stress factors. A total of seven DPs were detected in the different employed conditions as illustrated in Figure 6, which shows the decomposition route proposed based on *m*/*z* signals detected. The mass spectra are shown in Figure 7, where each *m*/*z* signal detected as ion molecular or ion product was considered to propose the correct elucidation. Table 3 shows the theoretical and experimental data of *m*/*z* signals acquired from MS analyzes, including additional information about molecular formula obtained from elemental composition calculator, which helped in better understanding about  the chemical structures and chemical routes associated.

**TABLE 3:** Illustrative table with description of theoretical and experimental *m*/*z* and molecular formula suggested for losartan and degradation products generated from forced degradation of losartan samples added and absent of nitrosamines. Experimental data obtained through LC‐MS analyzes in triple quadrupole system.

Compound	MS1	Theoretical mass (*m*/*z*)	Formula
Losartan (Xia et al. 2024)	423 [M+H]^+^	423 405 377 235 207 180	C_22_H_24_ClN_6_O^+^ C_22_H_22_ClN_6_^+^ C_22_H_22_ClN_4_^+^ C_14_H_11_N_4_^+^ C_14_H_11_N_2_^+^ C_13_H_10_N^+^
Losartan (experimental)	423 [M+H]^+^	423	C_22_H_24_ClN_6_O+
		391	C_21_H_20_N_5_O_3_^+^
		366	C_21_H_22_ClN_4_^+^
		341	C_17_H_15_ClN_5_O^+^
DP‐1	449 [M+H]^+^	449	C_23_H_23_ClN_7_O^+^
		419	C_22_H_21_ClN_7_^+^
		310	C_22_H_24_ClN_6_+
		282	C_17_H_14_ClN_2_^+^
DP‐2	447 [M+H]^+^	443	C_23_H_23_ClN_7_O^+^
		419	C_22_H_21_ClN_7_^+^
		310	C_22_H24ClN_6_+
		282	C_17_H_14_C_l_N_2_^+^
DP‐3	366 [M+H]^+^	366	C_21_H_22_ClN_4_^+^
		338	C_21_H_22_ClN_2_^+^
		282	C_19_H_25_N_2_^+^
DP‐4	338 [M+H]^+^	338	C_17_H_17_N_6_O_2_^+^
		310	C_16_H_17_N_6_O_2_^+^
		274	C_15_H_9_N_6_^+^
DP‐5	274 [M+H]^+^	274	C_15_H_9_N_6_^+^
DP‐6	391 [M+H]^+^	391	C_21_H_20_N_5_O_3_^+^
DP‐7	341 [M+H]^+^	341	C_17_H_15_ClN_5_O^+^

In order to have an easier interpretation about each DP suggested in this present study, these substances were discussed separately taking into account MS spectra (Figure 7) and the proposed degradation pathway (Figure 6).

##### 3.2.2.1 Degradation Product 1 (*m*/*z* 449) and Degradation Product 2 (*m*/*z* 447)

Degradation Product 1 (DP‐1) and Degradation Product 2 (DP‐2) were detected in degraded samples containing NAs in all degradation conditions tested with exception of basic medium. These substances can be generated from dimeric adducts formed between losartan molecules, followed by hydrolytic reaction and breakage of them (Figure 6). Making a chemical interpretation, both signals at *m*/*z* [M+H]^+^ 449 and [M+H]^+^ 447 can result from dimer adduct breakdown, followed by oxidation of alcohol group to aldehyde (*m*/*z* 447). The presence of these compounds in different samples, that is, generated under varied condition, means they are not uncommon and can be detected in routine during losartan stability studies. Losartan dimers are mentioned by [19, 20] in studies focused on drug degradation and characterization of novel substances.

In addition, the formation of these ion products occurs due to the partial breakage of the tetrazole ring moiety and subsequent electrophilic substitution reaction of residual nitrogen with the benzyl carbon from biphenyl ring (Smith and March 2001). The precursor ion at *m*/*z* 449 and *m*/*z* 447 is fragmented to ion product at *m*/*z* 419 through elimination of CHO. Subsequently, the *m*/*z* 419 ion is fragmented to *m*/*z* 359 (partial loss of tetrazole ring moiety), followed by cleavage of the benzylic carbon bond. This product ion at *m*/*z* 359 is fragmented to *m*/*z* signal 318 due to the loss of CH_2_N and dealkylation of butyl group attached to the imidazole ring. Therefore, DP1 and DP2 can be proposed as 2‐(2‐(4‐chloro‐5‐butlyl‐1H‐imidazol‐2‐yl)‐1‐phenyl‐1H‐imidazol‐5‐yl)ethanol and 2‐(2‐(4‐chloro‐5‐butlyl‐1H‐imidazol‐2‐yl)‐1‐phenyl‐1H‐imidazol‐5‐yl)carbaldehyde, respectively.

##### 3.2.2.2 Degradation Product 3 (*m*/*z* 366)

Degradation Product 3 (DP‐3) at *m*/*z* signal [M+H]^+^ 366 was detected as molecular ion or ion product depending on the sample. As a DP, it is suggested to be present on acid, basic and thermal degraded samples, even though this *m*/*z* signal is present in all conditions as well, being an important compound in the chemical route of degradation. In samples containing NAs this signal was also detected. The predicted theoretical mass and molecular formula were [M+H]*^+^ m*/*z* 366 and C_21_H_22_N_4_Cl^+^, respectively, as indicated by the elemental composition calculator. The proposed structure and fragmentation profile are shown in Figure 6. From precursor ion at [M+H]^+^ *m*/*z* 423 (losartan) it is proposed a partial fragmentation of the tetrazole ring, followed by the loss of (CH_2_O) attached to the imidazole ring. From [M+H]^+^ *m*/*z* 366 (DP‐3) the chemical route leads to *m*/*z* 338 and *m*/*z* 282. The signal at *m*/*z* 338 can be generated from a removal of the remaining tetrazole moiety (H_2_N_2_) through the cleavage of this fragment, while the signal at *m*/*z* 282 from the opening of the imidazole ring and the loss of mass (C_2_HCl). Xia et al. have mentioned the *m*/*z* signal at 366 as ion product from a specific DP at *m*/*z* 394 [20]. Thus, DP3 can be proposed as 2‐butyl‐4‐chloro‐1‐{[2′‐(diaziridin‐3‐yl)[1,1′‐biphenyl]‐4‐yl]methyl}‐1*H*‐imidazole.

##### 3.2.2.3 Degradation Product 4 (*m*/*z* 338) and Degradation Product 5 (*m*/*z* 274)

Degradation Product 4 (DP‐4) (Figure 6) was detected in degraded samples subjected to photolysis, oxidation, thermal and acid hydrolysis, regardless of whether or not they contained NAs. The signal [M+H]^+^ *m*/*z* 338 was predicted with a molecular formula C_17_H_17_N_6_O_2_^+^ (Table 3) being obtained directly from losartan (*m*/*z* 423) through the opening of the imidazole ring by oxidative hydrolysis. The ion product at *m*/*z* signal [M+H]^+^ 310 can be originated from cleavage and loss of CH_4_O (alcoholic chain). In some specific cases, such as degraded samples by acid hydrolysis in presence of NDIPA, it has been detected the *m*/*z* signal at 274 (Figure 6) as majority signal, indicating possibly a DP majority (Degradation Product 5, DP‐5), even with *m*/*z* signals at 338 (DP‐4) and 310 in lower intensity. Obviously, the chemical route proposed has included these substances as intermediate or final products (Figure 6). Two previous studies have described a DP at *m*/*z* signal 336, probably with an electronic rearrangement involving *m*/*z* signal at 338 [19, 20].

So, DP‐5 corresponds to the theoretical mass [M+H]^+^ *m*/*z* 274 and molecular formula C_15_H_9_N_6_^+^_._ When not the majority, the precursor ions are [M+H]+ at *m*/*z* 338 (DP‐4) and [M+H]+ *m*/*z* 310, which resulted from the loss of C_2_H_7_O_2_ and CH_7_O, respectively. Sequentially the loss of C_4_H_11_ possibly by hydrolysis results in degradation product DP‐5.

##### 3.2.2.4 Degradation Product 6 (*m*/*z* 391)

Degradation Product 6 (DP‐6) (*m*/*z* 391) was detected in samples subjected to thermal degradation, added of NDEA. It is important to consider this substance was also detected as ion product in other degraded samples subjected to UVA photolysis, oxidation, basic, and acid hydrolysis, being mentioned in the chemical degradation route proposed (Figure 6). From losartan (*m*/*z* 423), the stressing conditions promotes the opening of imidazole ring and structural rearrange by parallel oxidation to stabilize as [M+H]^+^ *m*/*z* 391, C_21_H_20_N_5_O_3_^+^. Other *m*/*z* signals (366, 340, and 313) were detected possibly from eventual fragmentation during analysis. One signal at *m*/*z* 340 might be generated from breakage of amidic group (Figure 6), easily eliminated from hydrolytic conditions. The current literature has mentioned DP‐6 *m*/*z* 391 as DP generated from exposure to ambient light or UVA‐photolysis [18].

##### 3.2.2.5 Degradation Product 7 (*m*/*z* 341)

Degradation Product 7 (DP‐7) (*m*/*z* 341) is represented as a compound structurally similar to DP‐4 (*m*/*z* 338) with minor differences observed from possible oxidation. This DP was detected in samples subjected to acid hydrolysis in the presence of NDBA. The *m*/*z* signal at 341 was also visualized as ion product in other MS spectra where other DPs appears as majority, in specific for stress degradation by oxidation, photolysis, thermal, and basic hydrolysis, in samples added of NDIPA. By acid hydrolysis, this signal was detected only for degraded samples absent of NAs. It is very important to consider that these signal as well as others discussed here come from losartan and multiple reactions possibly involved, which is natural and expected considering the complexity of stressed samples.

Like suggested for the DP‐4 (*m*/*z* 338), DP‐7 is generated directly from losartan (*m*/*z* 423) through the opening of the imidazole ring under acid hydrolysis and possibly oxidative reaction. However, minor modifications were observed in the chemical structure of DP‐4 possibly through oxidative reaction in C═O and NH groups, which led to be detected at *m*/*z* signal 341 (DP‐7).

### 3.3 In Silico Predictions

The Zeneth predictions performed in this study used the same set of processing constraints in order to maximize sensitivity and ensure consistent analysis across data sets. Table 4 shows the processing constraints and forced degradation conditions applied for this modeling. The parameters were chosen to allow the program to reasonably predict the greatest number of observed major and minor DPs under standard forced degradation conditions, while still providing access to more specific DPs.

**TABLE 4:** Number of degradation products obtained through in silico prediction using Zeneth software applied to degradation of losartan in different stress conditions, such as thermal, oxidative, photolytic, and hydrolysis (neutral, basic, and acidic).

Processes	Acidic/neutral/basic hydrolysis			Thermal		UVA	Oxidation	Total
**Number DPs (units)**	pH 1 (H^+^)	pH 7	pH 12 (OH^−^)	25°C	60°C	UV/vis	(H_2_O_2_)	Σ_somatory_
Losartan (API)	21	20	20	20	20	20	23	144
Losartan + NDMA	33	20	31	20	34	20	42	200
Losartan + NDEA	46	33	42	33	33	34	41	262
Losartan + NDIPA	46	33	42	33	33	33	42	262
Losartan + NDBA	46	33	42	33	33	33	41	261

Abbreviation: DP: Degradation product.

The score distribution allowed the assessment of the results using the LSL method, indicating that majority of first‐generation DPs were predicted based on experimental observations. All 1129 correctly predicted DPs received scores of ≥ 600. This indicates a high level of confidence in the chemistry behind these predictions. Among these, 985 DPs were predicted from conditions that included NAs individually with losartan, resulting in scores ≥ 800. Specifically for each NA studied, the number of DPs predicted were: losartan + NDMA—31 products at pH 12, 1 product with H_2_O_2_, 33 products at pH 1, and 1 product from thermal degradation; losartan + NDEA—1 product at pH 12, 1 with H_2_O_2_, 1 at pH 1, 1 from thermal degradation, and 1 from photolysis; losartan + NDIPA—1 product at pH 12, 1 with H_2_O_2_, 1 at pH 1, 1 from thermal degradation, and 1 from photolysis; losartan + NDBA—1 product at pH 12, 1 with H_2_O_2_, 1 at pH 1, 1 from thermal degradation, and 1 from photolysis. In the absence of NAs, the scores achieved were 600 ≥ scores ≤ 800.

Regarding the relationship between in silico findings and experimental data, some DPs predicted by Zeneth software matched those found by LC‐MS analysis: *m*/*z* 393, *m*/*z* 336, and *m*/*z* 310. In specific for the ion [M+H]^+^ *m*/*z* 393, this compound was detected as ion product in some analyses, despite not being considered a majority DP in this study. Chemically it results from imidazole ring moiety opening, oxidation, and conversion to nitrile and imide. Imidazole rings can be oxidized through cleavage by singlet oxygen to give imides and nitriles [18]. The imidazole ring undergoes a [4+2] cycloaddition with singlet oxygen, leading to an unstable endoperoxide which decomposes via [2+2+2] cycloelimination, yielding a nitrile and an imide. In relation to the signal [M+H]^+^ *m*/*z* 310 this compound is presented in the chemical route of degradation of DP‐4 (see Figures 6 and 7). Chemically, considering the chemical modifications proposed for that ions, alcohols react with acyl halides, acid anhydrides, mixed organic–inorganic anhydrides, imides, thioesters, N‐acyl azo‐aromatic compounds, and N‐acyl ammonium compounds to yield the corresponding esters. This reaction occurs very readily, especially in the presence of a basic media [38].

The Spartan 08 software package has been used to determine the theoretical quantum studies of the four NAs‐based compounds using DFT. Spartan 08 version was used for the molecular modeling computational study, and the software default settings were used to build all of the initial chemical structures. Three stages were used to optimize the geometry: MMFF94; AM1 was used for a fresh optimization; and DFT B3LYP/6.31G* (d, f) basis set level was used for a re‐optimization. Each NA was subjected to structural study, and DFT optimized the shape with the lowest potential energy, molecular electrostatic map and quantum descriptors including: LUMO energy (*E*_LUMO_), HOMO energy (*E*_HOMO_), HOMO–LUMO gap (Δ*E*_HOMO–LUMO_), ionization energy (IE), electron affinity (EA), chemical potential (*μ*), chemical hardness (*η*) chemical softness (*S*), electronegativity (*χ*), electrophilicity (*ω*) nucleophilicity (*N*), Δ*E*_back‐donation_, maximum charge transfer (Δ*N*_max_) [57].

DFT is used to compute the global reactivity descriptors of NAs (NDMA, NDEA, NDIPA, and NDBA) [58]. Following some references such as Perdew et al. [59] and the Janak theorem [60] the molecules electron affinity and ionization potential were calculated. Koopman theorem was also used to determine global reactivity for all reported molecules [61, 62]. Using the 6–311++G (d, f) basis set, global reactivity descriptors are calculated at the DFT/B3LYP level of theory (Table 5).

**TABLE 5:** Quantum reactivity descriptors calculated for four nitrosamines (NDMA, NDEA, NDIPA, and NDBA) using Spartan 08 software.

(eV)	*Ε*_HOMO_	*Ε*_LUMO_	Gap	IE	EA	(*μ*)	(*η*)	(*S*)	(*χ*)	(*ω*)	(*N*)	Δ*E*_b‐d_	Δ*N*_max_
NDMA	−8.46	1.25	−9.71	8.46	−1.25	−3.605	4.855	0.1030	3.605	1.338	0.747	1.214	−0.743
NDEA	−8.35	1.30	−9.65	8.35	−1.30	−3.525	4.825	0.1036	3.525	1.288	0.777	1.206	−0.731
NDIPA	−8.15	1.35	−9.50	8.15	−1.35	−3.400	4.750	0.1053	3.400	1.217	0.822	1.188	−0.716
NDBA	−8.25	1.19	−9.44	8.25	−1.19	−3.530	4.720	0.1059	3.530	1.320	0.758	1.180	−0.748

*Note*: IE = −*E*_HOMO_, EA = −*E*_LUMO_, gap (*E*_LUMO_ − *E*_HOMO_) or Δ(*E*_LUMO_ − *E*_HOMO_, *η* =  $\frac{\mathrm{IE}-\mathrm{EA}}{2}$; *μ* =  $-\frac{\left(\mathrm{IE}+\mathrm{EA}\right)}{2}$; *S* =  $-\frac{1}{2\eta }$; *χ* =  $\frac{\left(\mathrm{IE}+\mathrm{EA}\right)}{2}$; *ω* =  $\frac{\mu^{2}}{2\eta }$, Δ*E*_back‐donor_ =  $-\frac{\eta }{4}$, Δ*N*_max_ =  *ω*/ *η*.

Among all the NAs evaluated, NDMA has highest value of *η*, *ω*, and *χ*, NDIPA has highest value of *μ* and Δ*N*_max_, and NDBA has highest value of *S*. NDIPA transfers maximum charge in the direction of electrophile. The parameter Δ*E*_back‐donation_ has maximum value for the NDIPA which provides the useful information about reactivity of the molecular systems via electronic back‐donation process.

The Δ(*E*_LUMO_ − *E*_HOMO_) or gap energy was calculated based on established literature. The difference in energy between the HOMO and the LUMO can serve as a basic indicator of a compound chemical reactivity. A large energy gap between the HOMO and LUMO suggests that the compound is more stable and exhibits lower chemical reactivity. This is due to the energetically unfavorable process of adding an electron to the LUMO while simultaneously extracting an electron from the HOMO. In contrast a small gap suggests that the compound is more stable and displays higher chemical reactivity. Adding an electron to the LUMO and simultaneously removing an electron from the HOMO is an energetically favorable process [63].

**FIGURE 8:**
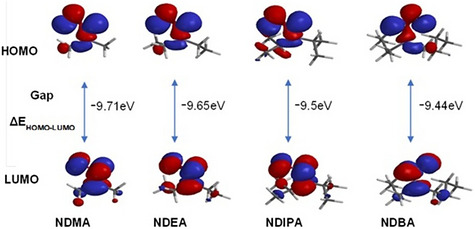
Lowest unoccupied molecular orbital (LUMO), highest occupied molecular orbital (HOMO), and energy gap of nitrosamines (NDMA, NDEA, NDIPA, and NDBA) illustrated graphically in 3D. Based on frontier molecular orbital technique. *Source*: Spartan 08 software.

These findings indicate that NDBA is relatively more reactive, with an energy difference of Δ*E* =  *E*_HOMO_ − *E*_LUMO_ = −9.44 eV. This has been observed experimentally in the UVA photolysis process during the quantitative determination of residual losartan in samples containing NAs using HPLC‐DAD and LC‐MS (Tables 1 and 2). The lower HOMO–LUMO energy gap of the NAs analyzed in this process, the lower the concentration of residual losartan, and therefore more reactive. Thus, the reactivity order for NAs is NDBA > NDIPA > NDEA > NDMA. In addition, the HOMO appears to be located around the oxygen atom, whereas the LUMO cloud, on the other hand, appears to be centered over the nitrogen atom and alkyl groups. This might be due to the nitroso group acting as an electron‐withdrawing agent. The Figure 8 shows the 3D molecular orbitals of NAs (NDMA, NDEA, NDIPA, and NDBA) according to their energy gap, HOMO, and LUMO based on frontier molecular orbital (FMO) technique.

**FIGURE 9:**
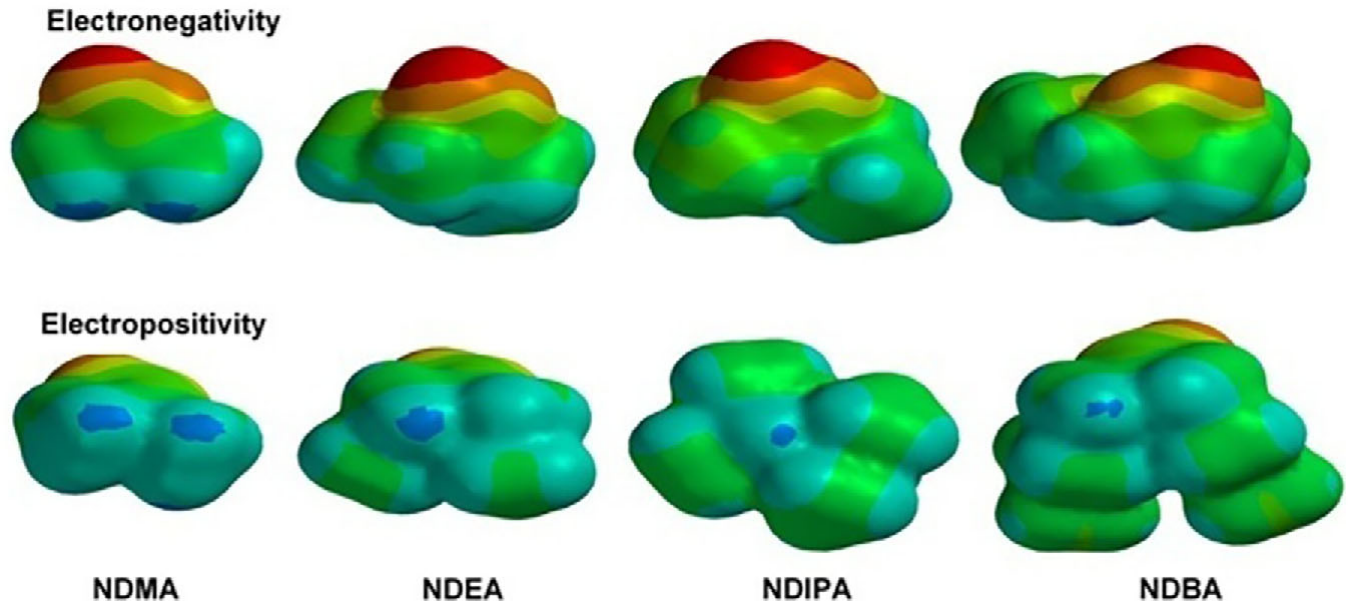
Molecular electrostatic potential (MEP) maps proposed for nitrosamines (NDMA, NDEA, NDIPA, and NDBA). *Source*: Spartan 08 software.

MEP maps are valuable tools for understanding molecular interactions, the behavior of chemical molecules, and their reactivity with other substances. In MEPs, regions are color‐coded to represent different electrostatic potentials: blue indicates positive potential areas, red means negative potential regions, and green represents neutral potential. Specifically, green surfaces reflect zero potential, red surfaces indicate negative potential values, and blue surfaces correspond to positive potential values [64]. The MEP map for the compounds NDMA, NDEA, NDIPA, and NDBA is shown in Figure 9. The DFT/B3LYP method is used to calculate the MEP maps of proposed compounds on the 6‐31G basis set. In addition, these data contain the MEP maps for the remaining chemicals.

The strong attraction between the proton and molecular surface points is indicated by the red surfaces on MEP, which correspond to high electron density. The blue surfaces of MEP represent the regions with the lowest electron density. Generally, the blue color regions of MEP are preferred sites for nucleophilic attack, whereas the red color regions are preferred sites for electrophilic attack. Understanding the reactive sites in a molecule is useful for further investigations into potential interactions in complex samples, such as those degraded by stress. In addition, this information can be complementary to the experimental data like observed here and can help the chemical interpretation related to drug degradation, chemical reaction, and decomposition chemical route.

## 4 Conclusions

The forced degradation of losartan during exposition to different aggressive agents caused significant decomposition, demonstrating this drug is chemically instable as already reported. The presence of NAs has influenced this behavior, there being different interpretations depending on the sample and degradation factor employed. Photodegradation by UVA was the agent less aggressive although clearly influenced by the presence of NAs, specially NDBA. Otherwise, the presence of NAs did not influence the thermal degradation process at 60°C. For the other degradation processes, the large data variability became difficult any definitive conclusions regarding the role of NAs, even though some important points of discussion were raised, such as the hypothesis about the protective effect or reactivity from the presence of NAs in samples. In addition, in silico assays based on databases of computational tools helped explain the results obtained analytically. Overall, the presence of NAs had a notable impact on the stability profile of losartan, where significant differences were observed comparatively between samples with or without NAs. In silico modeling by Zeneth and Spartan software were very useful in contributing to the understanding of the chemical influence and reactivity of NAs on degradation of losartan and the DPs formed.

## Author Contributions

**Paulo Roberto Rodrigues Martini**: writing – original draft, methodology, investigation, formal analysis, data curation. **Lilian Fanfa Machioli**: investigation, formal analysis. **Bruno Pereira dos Santos**: investigation, formal analysis. **Tiago Franco de Oliveira**: review and editing formal analysis, methodology. **Fávero Reisdorfer Paula**: review and editing, investigation, formal analysis, methodology. **Clésio Soldateli Paim**: original draft, formal analysis, methodology, supervision, conceptualization. **Andreas Sebastian Loureiro Mendez**: review and editing, Supervision, project administration, methodology, funding acquisition, formal analysis, conceptualization. All authors contributed equally to this work and approved the final version of the manuscript.

## Conflicts of Interest

The authors declare no conflicts of interest.

## Data Availability

The authors declare the data will be available on request from the authors.
